# Complete genome sequence of bean common mosaic virus from black bean in Vietnam

**DOI:** 10.1128/mra.00807-23

**Published:** 2023-12-20

**Authors:** Yutaro Neriya, Nami Kimpara, Tomohiro Suzuki, Huy Duc Nguyen, Hisashi Nishigawa

**Affiliations:** 1 School of Agriculture, Utsunomiya University, Utsunomiya, Tochigi, Japan; 2 Center for Bioscience Research and Education, Utsunomiya University, Utsunomiya, Tochigi, Japan; 3 Department of Plant Pathology, Faculty of Agronomy, Vietnam National University of Agriculture, Hanoi, Vietnam; Katholieke Universiteit Leuven, Leuven, Belgium

**Keywords:** bean common mosaic virus, potyvirus

## Abstract

This is a report of a complete genome sequence of bean common mosaic virus in Vietnam. This virus shares around 99% nucleotide identity with a Nepal isolate.

## ANNOUNCEMENT

*Bean common mosaic virus* belongs to the family *Potyviridae*, genus *Potyvirus,* and possesses a monopartite linear, single-stranded RNA genome. Bean common mosaic virus (BCMV) is transmitted by aphids, via seeds, and causes mosaic symptom on legume crops and serious damage of crop production in the world (1).

Some BCMV isolates were reported in Vietnam (1), but no complete genome sequence of Vietnam isolate was deposited to DDBJ/ENA/GenBank database.

In November 2019, we collected black bean leaves showing severe mosaic symptom in Ban Me Thuot, Dak Lak, Vietnam. Total RNA was extracted from collected black bean leaves using the Maxwell RSC Plant RNA Kit (Promega, WI, USA). For next-generation sequencing on the MiSeq (Illumina, USA), libraries were prepared with the KAPA Stranded RNA-Seq Library Preparation Kit (KAPA Biosystems, MA, USA) according to the manufacturer’s recommended protocol. The paired-end raw reads (301 bp) were cleaned using Trimmomatic (2) and assembled using SPAdes (version 3.9.0) (3). A BLASTn search using the nucleotide database (https://blast.ncbi.nlm.nih.gov/Blast.cgi) revealed that a contig of 10,034 nt showed high similarity to BCMV isolates; therefore, we next determined the rest of the virus genome. We used a commercial 5′ rapid amplification of cDNA ends (RACE) system (Invitrogen, CA, USA) to determine the terminal genome segments. We designed the BCMV-specific primers: VND-BCMV-GSP1 (5′-CTGTTGCGCCACCAATCT-3′) for first-strand synthesis and VND-BCMV-GSP2 (5′-GGCCCGCGCATCGTGCTGTG-3′) for dC-tailed cDNA synthesis. The amplified DNA fragment was purified by using NucleoSpin Gel and PCR Clean-up (MACHEREY-NAGEL, Germany) and cloned into a pGEM-T Easy vector (Promega, USA), and eight independent plasmids were sequenced using the BigDye terminator sequencing kit and the Applied Biosystems 3500 genetic analyzer (Thermo Fisher Scientific, USA). The 3′ region of the genome was amplified using PCR primers VND-BCMV-CP-F (5′-CAGATGCAGCTGAAGCATAC-3′) and PotyUni.M4(T) (5′-GTTTTCCCAGTCACGAC-dT(15) −3′), separated by agarose gel electrophoresis, purified by using NucleoSpin Gel and PCR Clean-up (Takara Bio, Japan), and sequenced directly using the VND-BCMV-CP-F primer. Sequences were assembled using SeqMan Pro version 12.3.1 (DNASTAR, Japan).

The complete nucleotide sequence is 10,063 nt (42.4% GC content) in length excluding poly(A) and encoded one open reading frame (9,669 nt) coding polyprotein of 3,222 amino acids, typical for potyviruses using ORFfinder (https://www.ncbi.nlm.nih.gov/orffinder/). A total of 6,472,166 reads were generated, resulting in 5,768 contigs, detected from the MiSeq analysis, and the coverage depth of the genome was 220.7. The BLASTn search using the nucleotide database showed that this virus had a high similarity of about 99% to a BCMV Nepal isolate. However, the 500 nt at the 5′ end region had a higher similarity to a Chinese isolate (KC832502) (94%) compared with the Nepal isolate (91%) suggesting that this strain might be a recombinant of two closely related viruses.

## Data Availability

The genome sequences of Vietnam isolate of BCMV were deposited at DDBJ/EMBL/GenBank database under accession number LC775775. The MiSeq data set was deposited in the NCBI Sequence Read Archive (SRA) under DRA accession number DRA017365 and SRA run accession number DRR511618.

## References

[1] Ha C , Revill P , Harding RM , Vu M , Dale JL . 2008. Identification and sequence analysis of potyviruses infecting crops in Vietnam. Arch Virol 153:45–60. doi:10.1007/s00705-007-1067-1 17906829

[2] Bolger AM , Lohse M , Usadel B . 2014. Trimmomatic: a flexible trimmer for Illumina sequence data. Bioinformatics 30:2114–2120. doi:10.1093/bioinformatics/btu170 24695404 PMC4103590

[3] Nurk S , Meleshko D , Korobeynikov A , Pevzner PA . 2017. metaSPAdes: a new versatile metagenomic assembler. Genome Res 27:824–834. doi:10.1101/gr.213959.116 28298430 PMC5411777

